# A targeted fluorescent nanosensor for ratiometric pH sensing at the cell surface

**DOI:** 10.1038/s41598-024-62976-2

**Published:** 2024-05-29

**Authors:** Charlotte Kromer, Aaron Katz, Ines Feldmann, Peter Laux, Andreas Luch, Harald R. Tschiche

**Affiliations:** 1 Product Materials and Nanotechnology, Department Chemical and Product Safety, German Federal Institute for Risk Assessment, Max-Dohrn-Str. 8-10, 10589 Berlin, Germany; 2 Institute of Pharmacy, Freie Universität Berlin, Berlin, Germany; 3 Material-Microbiome Interactions, Department Materials and the Environment, Federal Institute for Materials Research and Testing, Berlin, Germany

**Keywords:** pH sensor, pH, pHe, Nanosensor, Extracellular pH, Targeting, Nanoparticles, Biosensors, Imaging, Nanobiotechnology

## Abstract

The correlation between altered extracellular pH and various pathological conditions, including cancer, inflammation and metabolic disorders, is well known. Bulk pH measurements cannot report the extracellular pH value at the cell surface. However, there is a limited number of suitable tools for measuring the extracellular pH of cells with high spatial resolution, and none of them are commonly used in laboratories around the world. In this study, a versatile ratiometric nanosensor for the measurement of extracellular pH was developed. The nanosensor consists of biocompatible polystyrene nanoparticles loaded with the pH-inert reference dye Nile red and is surface functionalized with a pH-responsive fluorescein dye. Equipped with a targeting moiety, the nanosensor can adhere to cell membranes, allowing direct measurement of extracellular pH at the cell surface. The nanosensor exhibits a sensitive ratiometric pH response within the range of 5.5–9.0, with a calculated pKa of 7.47. This range optimally covers the extracellular pH (pH_e_) of most healthy cells and cells in which the pH_e_ is abnormal, such as cancer cells. In combination with the nanosensors ability to target cell membranes, its high robustness, reversibility and its biocompatibility, the pH_e_ nanosensor proves to be well suited for in-situ measurement of extracellular pH, even over extended time periods. This pH nanosensor has the potential to advance biomedical research by improving our understanding of cellular microenvironments, where extracellular pH plays an important role.

## Introduction

Cellular pH regulation is a fundamental aspect of cellular homeostasis and crucial for maintaining normal cellular function and survival. The precise control of intracellular and extracellular pH can be divided into the combination of two types of processes: (1) passive processes such as diffusion and selective ion channels and (2) active processes involving proton pumps, bicarbonate transporters and buffer systems, triggered by feedback mechanisms. The main purpose is to ensure that intracellular pH (pH_i_) levels remain within a narrow, tightly regulated range, typically between 6.8 and 7.2, depending on the cell type^1,2^. This is essential for maintaining the stability of numerous physiological processes, including enzyme activity, ion transport, and protein conformation. Therefore, intracellular pH regulation is a key factor influencing basic cell functions such as cell signaling, cell growth, and apoptosis^3^.

The extracellular pH (pH_e_) of the microenvironment surrounding cells is equally critical, both for the individual cell function but also for the functioning of tissues and organs as a whole. Aberrations in pH_e_ can impact cell–cell communication, metabolic processes, and even the immune response, contributing to the pathogenesis of numerous diseases^4,5^.

Extracellular pH imbalances can arise from various factors, including metabolic disorders leading to conditions like diabetic ketoacidosis, respiratory acidosis, or hypoxia-induced acidosis. Other pathomechanisms leading to tissue acidification are inflammation or carcinogenesis. While normal cells typically maintain a neutral pH_e_ (within the range of pH 7.2–7.4), the pH_e_ in tumor microenvironments is more dynamic^6,7^. The extracellular milieu in tumors often becomes acidic, with pH levels ranging from 6.2 to 6.9, primarily attributed to increased glycolysis and lactic acid production^8^. Studies have also indicated the presence of pH_e_ gradients and local pH_e_ heterogeneities within the tumor microenvironment^9,10^. These phenomena and the acidic microenvironment not only foster an invasive and metastatic tumor phenotype but also exert an influence on therapeutic resistance^11–14^. Therefore, accurate assessment of extracellular pH may help in evaluating the extent of tumor invasion, immune response and treatment strategies^15,16^.

Understanding the function and importance of extracellular pH regulation is essential for unraveling the intricacies of normal cellular and tissue function, as well as the pathogenesis of various diseases. Consequently, there is a pressing need for innovative and non-invasive methods that can provide real-time, high-resolution pH_e_ data for cells and tissues under physiological and pathophysiological conditions^17^.

While pH_i_ regulation has been extensively studied, the pH_e_ environment is often overlooked, possibly attributed to the substantial challenges associated with precise measurement of pH_e_ directly in cell’s proximity. Conventional methods, such as pH microelectrodes, are invasive, often lack the required spatial resolution as they only detect one point at a time and are not suitable for measuring local pH fluctuations in complex biological systems^18,19^. Nuclear Magnetic Resonance Imaging and Positron Emission Computed Tomography have also been reported for pH_e_ measurement^9,20^. These methods are limited by their high operation cost, low spatial resolution and their reliance on the distribution of the probes within the tissue of interest. Fluorescence-based techniques using fluorescent probes have the advantages of high sensitivity and excellent spatiotemporal resolution, as well as wide applications in 3D and even in vivo biosensing^21,22^. While conventional fluorescent dyes for sensing applications underlie limitations, like their susceptibility to fluctuations in excitation light intensity, changes in dye concentration and high background levels, ratiometric fluorescent sensors have been developed circumventing these limitations^21,23,24^.

Sensors based on fluorescent dyes^22^, proteins, lipids and nanoparticles^23,25,26^ share a common characteristic: their properties facilitate cellular uptake. However, concerning extracellular measurements this ease of cellular entry is also their primary drawback^6^. Consequently, most of them are better suited for intracellular rather than extracellular pH sensing applications^27^. One possible approach is the immobilization of the probe encased in thin films or gels^28,29^. This does enable pH sensing in the extracellular region but confines measurement to the interface where the gel adheres to the cell surface. However, accurately delivering extracellular pH data within three-dimensional cellular structures and in vivo settings presents an unresolved challenge. Therefore, an ideal and highly functional sensor would involve a sensor equipped with a targeting moiety, allowing precise localization on the cell membrane, the intended site for pH_e_ sensing. Nevertheless, there are only a few tools suitable for measuring the pH_e_ of adherently growing cells with high spatial resolution, and none of them is widely used in laboratories^30^. Consequently, the development of a cell-anchored ratiometric fluorescent sensor with high sensitivity emerges as a desirable solution for extracellular pH measurements.

This study describes the development of a highly functional pH_e_ nanosensor with a straightforward design concept utilizing readily available commercial components. The nanosensor is comprised of biocompatible polystyrene (PS) nanoparticles (NP), incorporating a reference dye in its core and a pH-responsive dye on its surface. For targeting the cell surface, the lectin wheat germ agglutinin (WGA) was utilized enabling the active targeting of the cell membranes and therefore the sensing of pH_e_ in close proximity to the cell. This versatile pH_e_ nanosensor has demonstrated the ability to perform non-invasive pH measurements within a pH range of 5.5–9.0, while preserving the integrity of the biological systems under investigation. The successful application of the pH_e_ nanosensor in three distinct eukaryotic cell lines (A549, BeWo, HaCaT) underscores its suitability to be utilized across diverse types of cell lines. Thus, a cell-anchored ratiometric fluorescent nanosensor with high sensitivity, quick response times and universal applicability was developed as a valuable tool for real-time monitoring of pH_e_. This pH_e_ nanosensor holds great potential for advancing the understanding of cellular physiology, disease mechanisms, and the development of targeted therapeutic interventions.

## Experimental section

### Materials and reagents

The solvents tetrahydrofuran (THF) and ethanol (EtOH) were purchased from Sigma-Aldrich (UV-spectroscopic grade) and used as received. The PS NP (100 nm) were purchased from Kisker Biotech and were ultrasonically treated for 10 min prior to use. The fluorescent dye Nile red (NR) and WGA labeled with fluorescein isothiocyanate (FITC) were purchased from Merck and employed without further purification. The cell culture materials and ingredients were purchased from Thermo Fisher Scientific, Carl Roth and Merck. All cell culture media components were purchased from Pan-Biotech (Aidenbach, Germany).

### pH_e_ nanosensor preparation

The pH_e_ nanosensor was prepared from commercially available carboxylated PS NP following a modified method which was previously described by Kromer et al.^31^. The PS NP had a size of 100 nm and a reported loading of carboxylic groups of 390 nmol/mg^32^. The reference dye NR was incorporated into the PS NP via a swelling procedure published by Behnke et al.^33^. In brief, 100 µL of a NR solution in THF (50 µmol/L) were added to 600 μL of an aqueous suspension of the PS NP (0.5 w%). After 30 min of shaking at RT, the suspension was centrifuged for 40 min at 16,000 g (5415D, Eppendorf). The supernatant consisting of unembedded NR dye was removed, followed by a washing step with 50 v% EtOH, a washing step with 10 v% EtOH and two washing steps with MilliQ H_2_O. Subsequently, the FITC-labeled protein WGA was conjugated to the NP. For that purpose, 1.2 mg of 1-Ethyl-3-(3-dimethylaminopropyl)carbodiimide and 2.4 mg of N-Hydroxysuccinimide in MES buffer were added to 300 µL NR-PS NP suspension (2.5 w%) and shaken for 30 min. After centrifugation at 16,000* g* for 40 min, the supernatant was removed and a subsequent washing step with MilliQ H_2_O was performed. Then, 300 µL of the FITC-labeled protein WGA in PBS (1 mg/mL) were added to the NP suspension and allowed to react for 3 h with continuous shaking. After another centrifugation step and removal of the supernatant, 120 µL of a 25 µM glycine solution were added and shaken for 30 min to block unspecific binding sites followed by three washing steps with MilliQ H_2_O. Finally, the concentration of the suspension was adjusted to 25 mg/mL before being stored in the fridge until further use.

### Particle size and zeta potential

The zeta potential and the particle size (hydrodynamic diameter) of the pH_e_ nanosensor were determined by dynamic light scattering (DLS) using a Zetasizer (Malvern Nano ZS, Malvern Panalytical). For particle size measurement and the determination of the polydispersity index (PDI), 2 µL of the NP suspension (25 mg/mL) was added to 1 mL MilliQ H_2_O in a Hellma quartz glass cuvette. The thermal equilibration time was set to 30 s at 25 °C. The intensity-weighted size distribution represents the average of three independent replicates, each consisting of three measurements with ten individual analyses. The dip cell kit (Malvern Panalytical) was used for the determination of the zeta potential. The NP dispersion was diluted as described above for the particle size determination. The zeta potential was determined as an average of three independent replicates, each consisting of three measurements with ten individual analyses. The particle size was also assessed using a transmission electron microscope (TEM) as described before^31^. 400 mesh 3.5 mm Formvar coated copper grids (Plano GmbH, Germany) were hydrophilized with 0.2% alcian blue (Sigma Aldrich, Germany) in 0.03% acetic acid solution. The grids were floated on alcian blue droplets for 10 min, and dried using a filter paper. The hydrophilized grids were used on the same day. 5 µL of a 5 mg/mL sample dispersion was applied on each grid, incubated for 1 min and the excess liquid was removed with a filter paper. Samples on the copper grids were observed in a Jeol 1400 Plus TEM (Jeol GmbH, Germany) operated at 120 kV. Material identification was done using diffraction pattern from published resources. Imaging was performed using a Veleta G2 camera (Olympus, Germany). Particle size was measured using iTEM software provided by Olympus. At least 4 different areas of each grid were examined per sample.

### pH dependent fluorescence measurements

Fluorescence spectra were recorded with a fluorospectrometer (LS 55, Perkin Elmer). Fluorescence measurements were carried out using an integration time of 0.1 s and slit widths for excitation and emission of 2 and 6 nm, respectively. To observe the pH-dependent fluorescence behavior of the pH_e_ nanosensor, 2 µL of nanosensor suspension (25 mg/mL) was added to 1 mL of buffer solution. The fluorescence spectra were recorded with excitation at 485 nm, with 21 different pH values ranging from 4.15 to 10.94, using the Britton-Robinson (BR) buffer in Hellma quartz glass cuvettes. The pH values of the BR buffer solutions were measured using a pH meter with an InLab Micro electrode (FiveGo F2 pH meter, Mettler Toledo GmbH). The pH meter was calibrated at 25 °C with standard buffers of pH = 9.21, 7.01, and 4.01 in a three-point calibration.

### Dye and protein loading

Dye loading was determined using a spectrophotometric method as previously described by Kromer et al.^34^. A calibration curve for the absorbance of NR in 50 v% THF in H_2_O at 530 nm was prepared. The concentration range of 1.25–15 nmol/mL was determined to be linear with R = 0.9985 using a linear regression model. As a second step, a defined volume of nanosensor suspension was added to an Eppendorf tube and centrifuged at 16,000* g* for 40 min. The supernatant was removed, and the NPs were dissolved in 500 µL THF and 500 µL H_2_O was added. The absorption at 530 nm was determined in a spectrophotometer (FoodALYT, Germany). The dye concentration was calculated as dye equivalents by using the calibration curve and used to calculate the dye loading per mg nanosensor.

For the determination of the protein loading (equiv. of WGA per mg particle) a modified indirect Coomassie assay was performed^35^. The NP suspensions were prepared by adding 20 µL NP suspension (0.5 mg NP) to 880 µL MilliQ H_2_O and 100 µL of Coomassie blue concentrate (Protein Assay Kit, Bio Rad). As a control 20 µL of NP without lectin were prepared in the same manner. The mixture was incubated for 10 min and then centrifuged at 16,000* g* for 40 min. The supernatant, containing unbound Coomassie blue, was collected and 50.4 µL of BSA solution (8 mg/mL in MilliQ H_2_O) was added to 700 µL supernatant. 100 µL of the resulting solution was transferred to a 96-well plate in triplicates. Titration of the Coomassie blue was performed by adding different volumes of Coomassie blue (0, 1, 5, 10, 50, and 100 µL) to 700 µL of MilliQ H_2_O in Eppendorf tubes, followed by the addition of 50.4 µL of the BSA solution. Triplicates of each sample were transferred to the same 96-well plate. Similarly, BSA titration was carried out by setting up a standard series of Coomassie blue. 10, 20, and 50 µL of Coomassie blue were made up to 700 µL with MilliQ H_2_O and variable amounts of BSA (0, 1, 2, 4, 6, 8, 10, or 15 µL) were added. Triplicates of each sample were then transferred to the second 96-well plate. For both plates the absorbance at 595 nm was measured using a plate reader (BioTek Synergy Neo 2, Agilent Technologies). The amount of conjugated lectin as BSA equivalents was calculated.

### Cell culture

The human lung epithelial cell line A549 and the human placenta cell line BeWo were purchased from ATTC (American Type Culture Collection). The human immortal keratinocyte cell line HaCaT was purchased from DSMZ (German Collection of Microorganisms and Cell Cultures GmbH). All cells were cultivated in 5% CO_2_ at 37 °C and grown in DMEM medium, containing 10 v% FCS for A549 and BeWo and 5 v% for HaCaT, 100 U/mL penicillin, 100 mg/mL streptomycin, and 2 mM L-glutamine.

### Cell viability

To assess the viability of the cells after incubation of the NP the colorimetric viability assay kit WST-1 (Roche Diagnostics GmbH) was used. Here, the amount of formazan dye formed is directly related to the metabolic activity of cells. The assay was carried out as described in the manufacturer’s instructions. In brief, cells were seeded at 2 × 10^4^ cells/well in transparent 96-well plates and incubated with the NP for various time periods. After addition of 10% WST-1 reagent and incubation for 30 min, the absorbance was measured at 450 nm (620 nm was used as reference wavelength) in a multiplate reader (BioTek Synergy Neo 2, Agilent Technologies). To account for potential absorbance of the NP at 450 nm, the absorbance of each well was also measured before addition of the WST-1 reagent and subtracted from the final value. Results are reported as relative WST-1 activity, where 100% corresponds to the absorbance measured in control cultures and 0% to the dead-control treated with 1% Triton-X for 30 min.

### Cell binding assay

To evaluate the cell adhesion of the WGA-conjugated nanosensor, binding studies were performed on the 3 cell lines A549, HaCaT and BeWo. For this purpose, the suspended cells were incubated for 5 min at 37 °C with WGA-conjugated NP at 1.25 mg/mL in Eppendorf tubes. NP without WGA served as a control. After the incubation, the cells were centrifuged at 300 *g* for 6 min. The supernatant containing the unbound NP was removed. The cell pellet was resuspended in cell culture medium, transferred to a 96 well plate, followed by measurement of the NP fluorescence (NR, 530 nm) in a plate reader. Cells with NP without centrifugation served as 100% control for calculating the relative amount of NP bound to the cells.

### Scanning electron microscopy (SEM) imaging

For SEM imaging, A549 and HaCaT cells were grown on 1 × 1 cm glass slides and incubated for 10 min with 0.825 mg/ml pH_e_ nanosensor. The samples underwent three washing steps with PBS and were then fixed with 2% glutaraldehyde for 2 h. After three more washing steps with PBS, the cell specimens were dehydrated using a graded series of alcohol (30%, 50%, 70%, 90%, and 99% ethanol). Liquid carbon dioxide was used as a transitional fluid for critical point drying (EM CPD300, Leica, Germany). The cell specimens were sputter-coated with a 30 nm conductive gold layer (EM ACE600 table-top coater, Leica, Germany) and examined with an environmental scanning electron microscope (XL 30 ESEM, FEI, Netherlands) equipped with a secondary electron detector and operated at an electron accelerating voltage of 25 kV. At least three random sections per sample were analyzed.

### CLSM imaging

All cells were live cell imaged in Ibidi µ-dishes at 37 °C using a confocal laser scanning microscope (LSM 700, Carl Zeiss Jena GmbH, Jena, Germany). A 63 × /N.A. 1.4 objective with oil immersion was used for imaging. XY images were acquired with 1024 × 1024 pixels. The standard laser and filter set for FITC and NR were used. Cells without nanosensor were imaged in the same way to determine background signals and autofluorescence. The imaging settings, such as laser intensity, gain, and contrast, were first optimized, saved, and then used for all imaging procedures to ensure comparability.

### Image analysis and pH calibration

As describe above, all images were acquired with identical microscope settings. No background correction was performed as autofluorescence was not observed under the chosen measurement conditions. For the image analysis the fluorescence intensities (FI) of the FITC and NR channel of the whole image were calculated with ImageJ. The FITC FI divided by the NR FI yields the total FI ratio of the pH_e_ nanosensor. A calibration curve was generated using BR buffers with 11 different pH values ranging from 4.15 to 10.05. For imaging, the cells were washed twice with PBS before adding the nanosensor at a concentration of 0.825 mg/mL. After incubating the nanosensor for 10 min, the supernatant containing unbound nanosensor was removed. Subsequently, the cells were washed with BR buffer of the pH of interest and imaged. This procedure was carried out in three independent triplicates for all three cells lines and all 11 buffers. To obtain the calibration curve, a four-parameter sigmoidal curve was fitted to the pH calibration data using Graph Pad Prism. A reverse pH estimation can also be performed with this function, allowing for determination of unknown pH values from the FI ratio of an image.

### Statistics

Statistical analysis was carried out with GraphPad Prism 9. All data are presented as mean ± standard deviation and were acquired in triplicates. Groups were compared using the t-test and one-way ANOVA. *P* values ≤ 0.05 were considered statistically significant.

## Results and discussion

### Fabrication of the pH_e_ nanosensor

**Figure 1:**
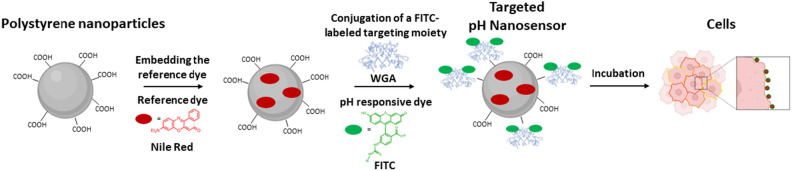
Schematic illustration of the pH_e_ nanosensor fabrication starting from a functionalized PS NP. The reference dye NR is embedded into the particle by a swelling procedure. The lectin WGA labeled with the pH-responsive dye FITC is conjugated to the NP as targeting moiety therefore enabling the pH_e_ nanosensor to target the cell membrane of eukaryotic cells.

For the preparation of the pH_e_ nanosensor, a straight forward two-step strategy was carried out as shown in Fig. 1. First, the reference dye NR was embedded into PS NP by a previously established swelling method^33,34^. The PS NP were chosen as the platform for the pH_e_ nanosensor, as they are readily available with different surface functionalizations. Here, NP with carboxylic surface groups were chosen, as they are hydrophilic and allow conjugation of dye molecules as well as proteins. These negatively charged PS NP are biocompatible and stable in cell culture media^36,37^. As a second step, the protein WGA, labelled with the pH-responsive dye FITC, was covalently attached to the carboxylic groups of the PS NP surface via carbodiimide coupling. WGA is a lectin, which is a carbohydrate-binding protein that specifically binds to N-acetylglucosamine and sialic acid residues. WGA conjugated to fluorophores are frequently used to label and identify specific glycoproteins or glycolipids on cell membranes in microscopy studies^38,39^. Here, the WGA was applied to facilitate the targeting of the cell membranes by the pH_e_ nanosensor. The protein loading was optimized, with respect to optimal binding of the NP to the cells and optimal signal intensities of FITC (dye and protein loading data in SI, Table A1). For ratiometric fluorescence sensing of pH in the visible wavelength region, hydrophobic red emissive NR was chosen as a pH-inert reference dye and hydrophilic and biocompatible FITC as a pH-responsive dye^31,40^. Upon incorporation into the NP, NR showed a homogeneous particle loading without leakage in aqueous dispersions, high photochemical stability and brightness. FITC reveals a strong green fluorescence solely at basic and neutral pH values with an optimal working range for pH sensing for working in eukaryotic cells. The dyes exhibit spectrally discriminable emission bands and can be read out with a standard CLSM setup using standard lasers and filter settings. While FITC exhibits its excitation maximum at approximately 490 nm, NR, on the other hand, has its excitation maximum at 530 nm. Notably, the excitation range of NR is broad and overlaps with the excitation maximum of FITC. Therefore, both dyes can be excited at the same wavelength, but the emission can be read out at different wavelengths, enabling ratiometric pH measurement with only one excitation wavelength. This simplifies the equipment needed to do measurements, and thus there are less error sources. The chosen ratiometric design concept allows a correlation of the calculated intensity ratios of the pH_e_ nanosensor FITC and NR fluorescence with pH neglecting local concentration differences of the sensor. Hence, the pH_e_ nanosensor is a useful tool for pH detection that can be used in a standard laboratory environment under a wide range of conditions. Despite its efficient performance, there is no need for advanced techniques or materials other than a CLSM. In this study, functionality and practicality was prioritized over complexity and novelty, as this provides the greatest benefit to the ever-growing need for easy and ready-to-use methods.

### Characterization of the pH_e_ nanosensor

**Figure 2:**
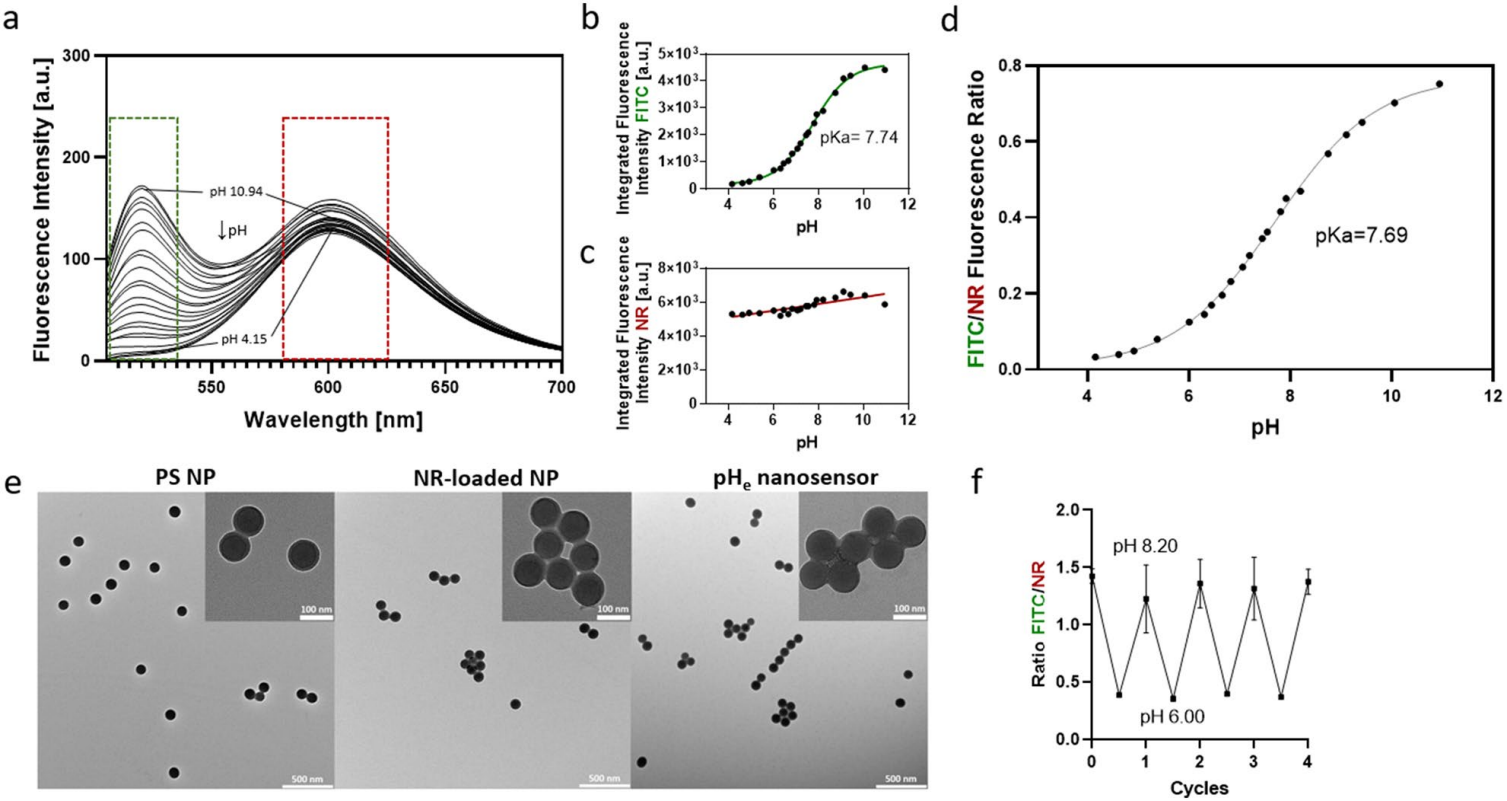
Characterization of the pH_e_ nanosensor. (**a**) Fluorescence spectra of the pH_e_ nanosensor excited at 485 nm in different buffers with known pH. (**b**) Sigmoidal fit of the integrated FI of FITC (green box = area of signal integration) plotted against the pH value of the respective buffer. (**c**) Integrated FI of NR (red box = area of signal integration) plotted against the pH value of the respective buffer and derived pKa value. (**d**) Ratio of the integrated FI of the FITC and the NR emission plotted against the corresponding pH with sigmoidal curve fit. (**e**) TEM images of the precursor PS NP (left), the NR-loaded NP (middle), and the pH_e_ nanosensor consisting of NR-loaded NP conjugated to WGA-FITC (right). (**f**) pH reversibility study of the pH_e_ nanosensor between pH 6.0 and 8.2.

The precursor PS NP size was determined to be 114.1 ± 3.9 nm through DLS and 94.0 ± 8.5 nm through TEM, a result that matches the value of 100 nm provided by the manufacturer (Table 1). The size of the particles remained unchanged after incorporation of NR dye and the hydrodynamic diameter increased only slightly after the conjugation of the protein WGA. The particles have a spherical morphology and low aggregation behavior, which also remains unchanged after functionalization of the particles (Fig. 2e). The consistency of particle size and morphology is critical as it excludes the aggregation of particles and thus ensures the sensor's reliability and performance^41^. Furthermore, the small size of the particles allows local pH imaging with high resolution. This is in contrast to other particulate sensors, which have larger particles in the µm range, hindering the ability to image and detect pH at a cellular resolution^29^. The zeta potential analysis revealed a negative charge of approximately − 63 mV and − 66 mV for the precursor NP and the NR-loaded NP, respectively. This indicates a high stability of the suspension, as suspensions with zeta potentials more positive than + 30 mV or more negative than − 30 mV are considered to have a high colloidal stability maintained by electrostatic repulsion^42–44^. Furthermore, after WGA-FITC conjugation, while the zeta potential increased, it remained negative at around − 31 mV. Importantly, the critical properties for suspension stability, namely size and zeta potential remain stable even after functionalization, underscoring the suitability of the nanosensor for the imaging of pH_e_ in cells.

**Table 1:** Comparison of the particle size and zeta potential of the precursor PS NP with the NR-loaded NP and the pH_e_ nanosensor by TEM and DLS.

	Size (TEM) [nm]	Size (DLS) [nm]	PDI (DLS)	Zeta potential [mV]
PS NP	94.0 ± 8.5	114.1 ± 3.9	0.012	− 62.7 ± 1.1
NR-loaded NP	92.2 ± 9.7	113.8 ± 2.6	0.018	− 66.0 ± 5.3
pH_e_ nanosensor	86.5 ± 9.2	125.8 ± 6.5	0.050	− 30.6 ± 1.4

### Fluorescence characterization

The fluorescence properties of the pH_e_ nanosensor were examined at different pH values in BR buffer to assess its ability for pH determination in cellular systems. As shown in Fig. 2a, the pH_e_ nanosensor is excited at 485 nm and exhibits a strong fluorescence signal with maxima of NR at 600 nm and FITC at 520 nm under neutral and basic pH conditions. The pH-dependent fluorescence measurements confirmed that the FI of FITC correlates with changes in pH while the FI of NR is pH-independent. The FITC fluorescence signal is highest at pH 10.05, decreases upon acidification, and eventually disappears at pH ≤ 4.5. Integrating the FI of FITC in the peak area and plotting against pH reveals a sigmoidal behavior  (Fig. 2b), whereas that of NR remains constant (Fig. 2c). The plot of the ratio of the integrated FI of FITC and NR as function of pH (Fig. 2d) derives a pKa value of 7.69 that is slightly shifted to basic pH values compared to FITC conjugated only to WGA with a pKa value of 6.7. This shift is attributed to the coupling of FITC to a negatively charged particle^24^. However, due to it being comparatively small no changes in protein structure and activity are expected. The working range of pH 5.5–9 is rather broad when compared to other pH sensors, due to the relatively flat slope of the curve. Thus, the working range of the nanosensor for pH detection is well suited for fluorometric pH sensing in the physiological pH range of eukaryotic cells.

The reversibility of the pH sensing capabilities of the pH_e_ nanosensor was tested by imaging the FI ratio of FITC to NR at different pH in the CLSM. After repetitive changes of the extracellular pH between 8.2 and 6.0, the FI ratio of the pH_e_ nanosensor was not affected (Fig. 2f). Therefore, leaching of the dyes from the sensor and bleaching of the dyes can be excluded, as this would change the FI ratio over time. This reversible response is a prerequisite of pH_e_ nanosensors to be utilized for tracking continuous pH fluctuations in biological systems over extended periods of time. Additionally, the long-term stability of the pH_e_ nanosensor at different pH values from 4.61 to 9.31 was investigated over 24 h (SI, Fig. A1). The FI intensity ratios remained constant, indicating no loss of function even at extreme pH value and long investigation times.

### Screening target cell lines

**Figure 3:**
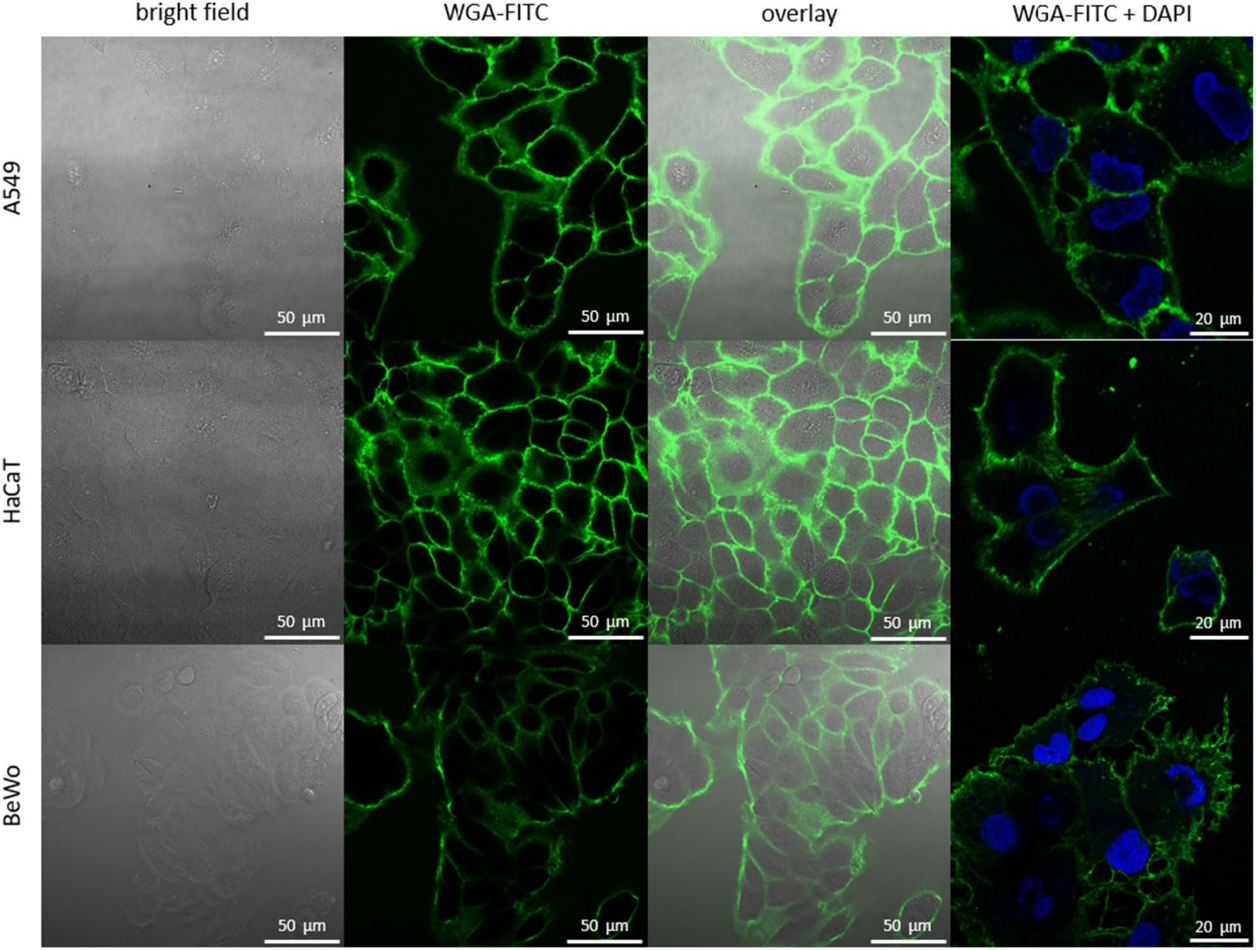
Screening of targeting capabilities of WGA as a targeting moiety. CLSM images of the lung cell line A549, skin cell line HaCaT and placenta cell line BeWo incubated with 5 µg/mL WGA-FITC (green) and Hoechst for nuclei stain (blue).

In order to measure pH_e_, it is necessary for the pH_e_ nanosensor to be in close proximity to the target cells. For this purpose, the pH_e_ nanosensor was conjugated to the cell membrane targeting protein WGA, which enables the attachment of the pH_e_ nanosensor to the cell surface. To first evaluate the suitability of WGA as a cell membrane targeting component, three different cell lines from different tissues were incubated with FITC-labeled WGA and examined with CLSM (Fig. 3). Here, the selection of A549 (human lung epithelial cells), BeWo (human placenta cells), and HaCaT (human skin keratinocytes) aimed to ensure tissue diversity. This selection was made with the understanding that the pH_e_ is highly relevant across different tissues in an organism. As it can be seen in the CLSM images, WGA exclusively labels the cell membranes of all 3 cell lines without entering the cells. This proves its suitability as a targeting moiety for the cell surfaces of various cell types, enabling the utilization for measuring the pH_e_.

**Figure 4:**
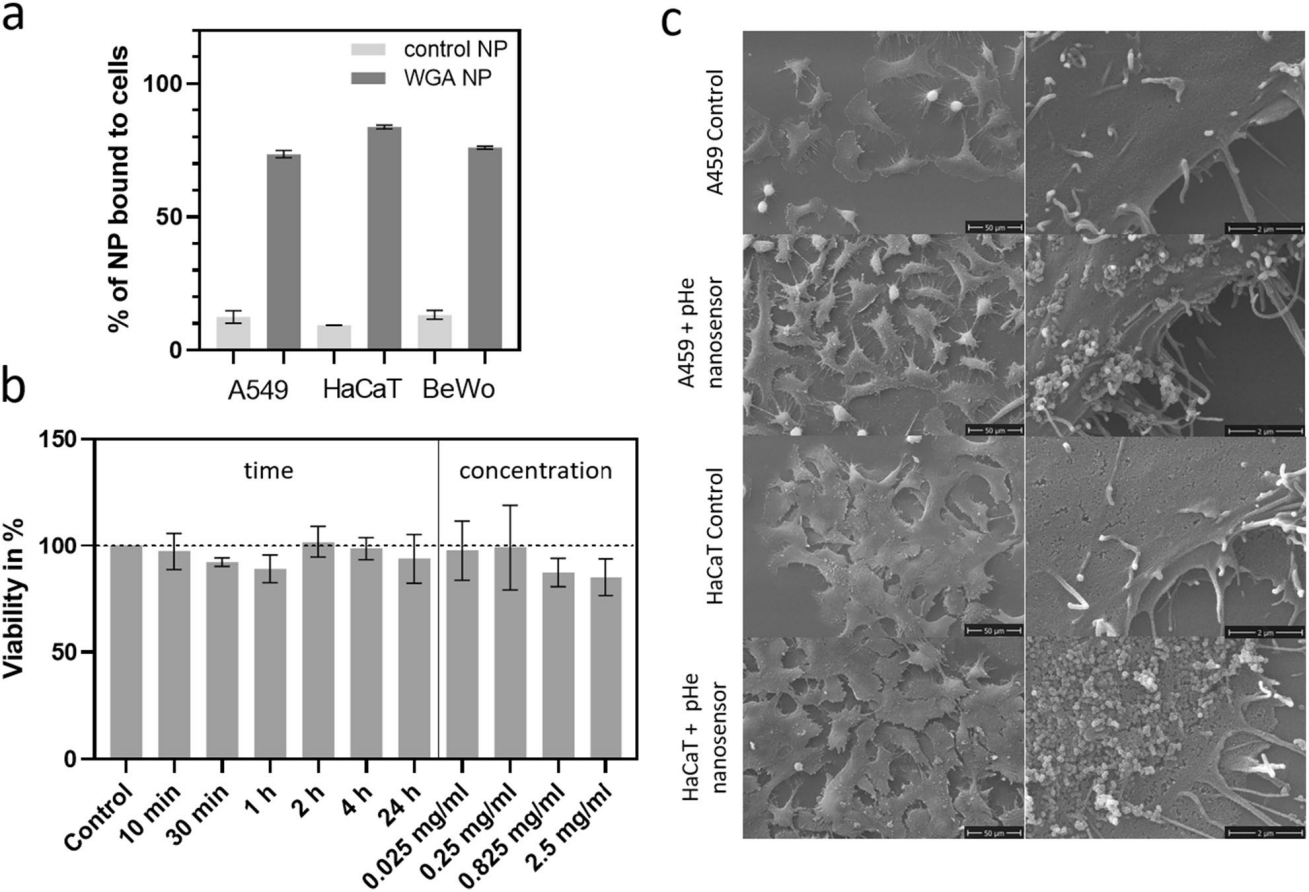
(**a**) pH_e_ nanosensor cell binding assay with WGA-conjugated nanosensor (WGA NP) and precursor nanosensor without WGA as non-targeted control NP (control NP). The amount of NP bound to the cells was quantified via the NR fluorescence signal and correlated to the total amount of NP (100%). (**b**) Viability of A549 cells after exposure of the pH_e_ nanosensor for different time points and different concentrations related to the control without pH_e_ nanosensor (**c**) SEM images of A549 and HaCaT cells. Samples without (control) and with the pH_e_ nanosensor (0.825 mg/ml). Additional SEM images can be found in the Supplementary Information in Fig. A2.

To assess the effectiveness of WGA as a targeting moiety when conjugated to the NP, a cell binding assay was performed, quantifying the amount of NP that bind to the cells with and without conjugated WGA (Fig. 4a). For this purpose, suspended cells of the 3 cell lines were incubated with WGA-conjugated nanosensor (WGA NP) and the precursor NP before WGA labeling was used as a non-targeted control sample (control NP). After incubation, the cells were centrifuged, and the supernatant containing unbound NP was removed. The cell-bound NP were quantified via the fluorescence signal of the embedded NR in the NP and were related to the total fluorescence signal of the NP before centrifugation. The largest fraction of the control NP was removed by centrifugation, and only approximately 10% of the particles were found in the cell pellets. This might be due to electrostatic affinity or other unspecific bindings of the NP to the cells. For the WGA NP, approximately 75–85% of the particles were found to be bound to the cells, indicating that a large fraction of the WGA NP binds to the cells via specific protein binding. These numbers align with literature values of BSA-WGA binding to, e.g., Caco-2 cells^45^. This indicates that the activity of WGA to act as a targeting moiety in conjunction with the NP is not compromised when the protein is conjugated. To further characterize the targeting properties, the cells were investigated with SEM after incubation with the pH_e_ nanosensor (Fig. 4c, additional SEM images in the SI, Fig. A2). The images revealed that the spherical nanosensor binds to the cell membrane, creating a sensor layer that uniformly covers the cell surface. This uniform distribution is crucial for obtaining strong signal intensities at the area of interest in fluorescence experiments and accurately measuring local pH levels.

To utilize the nanosensor as a tool for pH_e_ sensing in in vitro applications such as cell cultures, tissues or organoids, it is important that the pH_e_ nanosensor does not affect the target cells or their viability. Despite the selection of the components used for the pH_e_ nanosensor being based on components to be known as non-toxic in the concentrations used, a cell viability assay was performed. Here, a WST-1 assay was selected to assess whether exposure of the pH_e_ nanosensor influences the cell viability (Fig. 4b). Different concentrations and incubation times were tested, ranging from 10 min to 24 h, to cover a variety of application times sufficient for imaging experiments. It was found that no concentration up to the tested maximal concentration of 2.5 mg/mL and none of the incubation times tested showed a significantly toxic effect on the cells. Additionally, no morphological changes were observed in the cells in the SEM images, compared to the control (Fig. 4c, additional SEM images in the SI, Fig. A2). Therefore, the pH_e_ nanosensor was found to be cell-compatible, enabling its safe application, particularly for brief time periods of several minutes or hours in cell culture experiments.

Ideally, pH_e_ nanosensors should function with minimal impact on cellular processes to avoid disrupting the natural cell environment. Some studies have shown local pH_e_ measurements with high resolution through the expression of fluorescent sensor molecules by the target cells^46,47^. Although this method is highly effective, the expression of fluorescent dyes represents a significant intervention in cell physiology. To maintain a cell’s natural state during imaging, it is preferable to opt for minimally invasive pH sensing methods. Anderson et al. developed a pH_e_ measurement tool by attaching a dye to a low-insertion protein that inserts into the cell membrane^48^. The study yielded precise surface pH values with relatively little impact on the cell function. However, local pH determination at the cellular level, which is the ultimate goal of surface pH determination, especially in environments with pH heterogeneities, was lacking.

### Imaging of the pH_e_ nanosensor and determination of pH_e_

**Figure 5:**
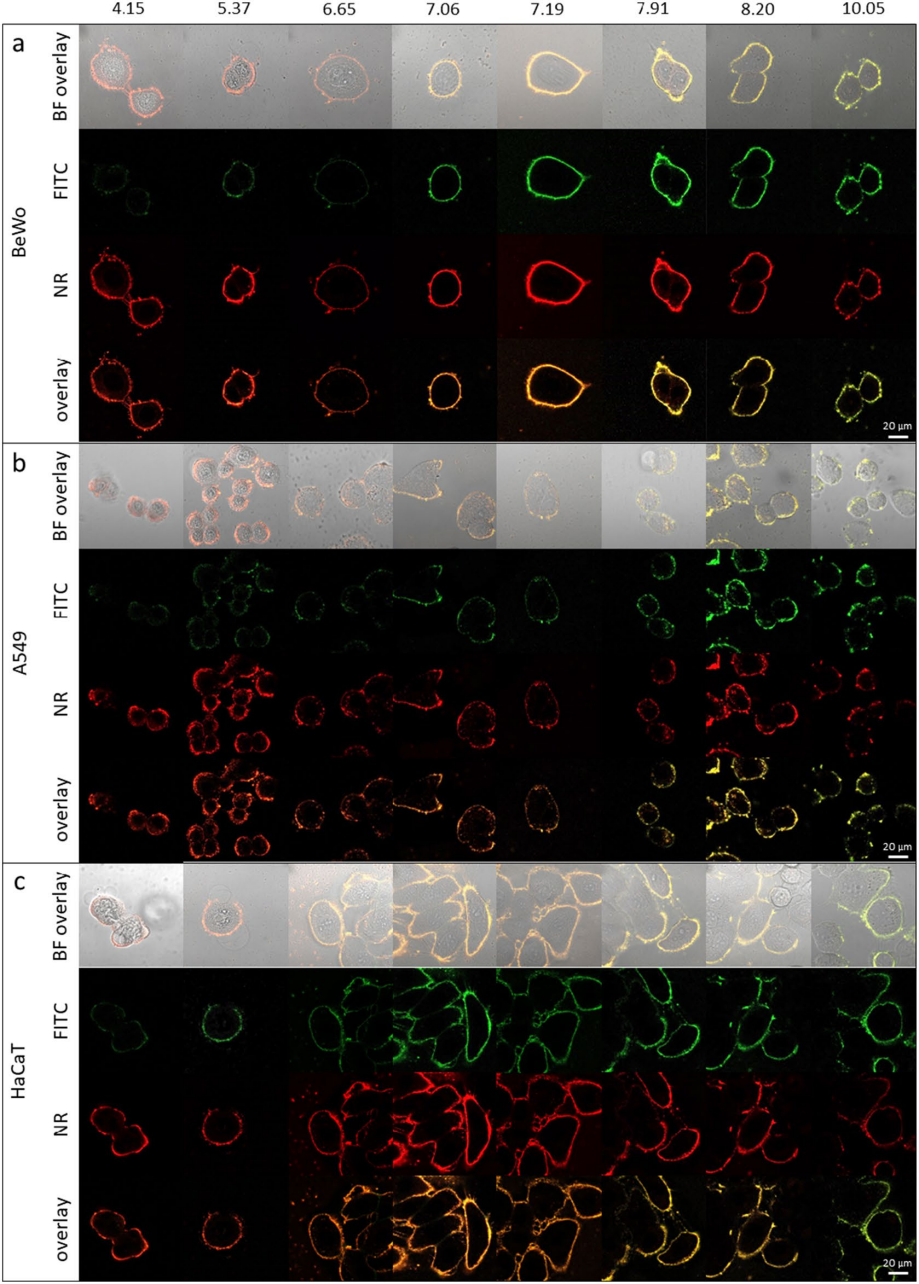
CLSM images of the 3 different cell lines (**a**) BeWo, (**b**) A549 and (**c**) HaCaT with the pH_e_ nanosensor. The cells were imaged in 8 different buffers with pH values from 4.15 to 10.05. Displayed are the FITC (green) and NR (red) channels, the fluorescence overlay and the bright field channel with fluorescence overlay. The 20 µm scale bar applies to all images.

To investigate the pH_e_ nanosensors functionality and robustness in cellular applications, its capability to determine the pH on the cell surface was investigated. For this purpose, the three different cell lines were incubated with the pH_e_ nanosensor and imaged with CLSM in different pH buffers with pH values from 4.15 to 10.05 (Fig. 5). Here, the fluorescence signals of the pH_e_ nanosensor were found to localize at the cell membranes, as it can be seen in the overlay images of the BF, the NR (red) and the FITC (green) channel. This shows effective targeting of the cell membranes of all three kinds of cell lines by the pH_e_ nanosensor, ultimately enabling the direct sensing of pH_e_ on the surface of the cell. Furthermore, the co-localization of the FITC and NR fluorescence signals shows that both dyes are located at the pH_e_ nanosensor, and that the dyes did not leak but are stable, hence allowing ratiometric determination of pH_e._

**Figure 6:**
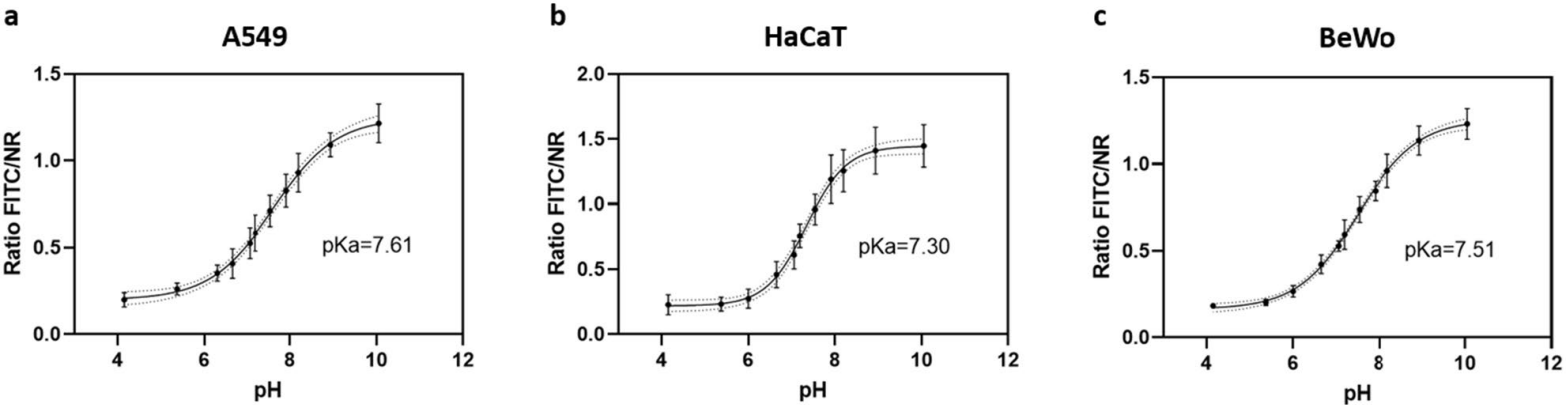
The calibration curves derived from the CLSM experiments in the pH range from 4.15 to 10.05 with sigmoidal curve fit in the three cell lines (**a**) A549, (**b**) HaCaT and (**c**) BeWo and the respective derived pKa values.

When imaging the pH_e_ nanosensor and the cells in buffers with known pH, the FI of FITC does respond according to the pH value with high FI in basic pH and low FI in acidic pH. This also can be seen in the overlay of the FITC and NR fluorescence channels showing the ratio of the two channels as a color change from red to yellow for acidic to basic pH. The mathematical realization of this overlay is the calculation of the FI ratio, by dividing the FITC FI through the NR FI. This ratiometric concept allows a correlation of the calculated FI ratio with pH, neglecting local concentration differences of the pH_e_ nanosensor. Plotting the FI ratio against each corresponding pH, yields a calibration curve for each individual cell line tested (Fig. 6). Notably, the pH curves and pKa values observed in the cell culture experiments align with those determined in the acellular assay, highlighting the pH_e_ nanosensor’s robustness. The pH_e_ nanosensor’s functionality is not compromised by the biological system despite variations in experimental conditions, including salt concentrations, protein interactions, matrix effects, and cellular autofluorescence. This robustness is essential to affirm the sensor's suitability for biological applications, demonstrating its ability to remain functional even in complex biological environments.

The advantage of the pH_e_ nanosensor is its remarkably simple experimental procedure for labeling cell surfaces and therefore the target cell line. In contrast to some sensors that need to be genetically encoded to be expressed on the cells surface^46,47^ or extensively incubated (several hours to overnight)^40^, the pH_e_ nanosensor’s application only required a single step: just adding it to the cell culture medium for 10 min. No additional washing step is required for imaging, as shown in Fig. A3. The excellent stability of the pH_e_ nanosensor over a wide pH range and for long measurement times, combined with its cell compatibility and ease of use, make it an ideal tool for pH_e_ measurements. Regarding the simplicity of the labeling procedure, a PEG-FITC-based probe by Ohgaki et al.^49^ is comparable to the pH_e_ nanosensor. A drawback of the aforementioned probe is its narrow pH measuring range of approx. 5.5 to 7.0. Chen et al.^50^ also report a sensor with a similar limited pH measuring range of 5.9 to 6.4. Such a narrow pH range reduces a sensor’s applicability to very specific areas of interest. Nevertheless, their sensor system exhibits great potential for pH_e_ measurement through anchoring a lipid to the cell membrane.

## Conclusions

In this study, a ratiometric pH_e_ nanosensor for the determination of extracellular pH was developed. The measurement of pH_e_ with this nanosensor is fast, robust, fully reversible, and the nanosensor showed no leaching of dyes even after long periods of time. By conjugating the nanosensor with a protein that binds to cell surfaces, the pH_e_ nanosensor can rapidly target different cell lines of interest such as lung, skin and placenta cells. As demonstrated by CLSM and SEM experiments, the active targeting approach proves to be highly effective, thus enabling the pH_e_ nanosensor to accurately measure pH levels on the surface of cells. This approach enables a very precise and locally resolved measurement of the pH_e_ while being cell-compatible and universally applicable in cell lines of different tissues.

In the future, this pH_e_ nanosensor can further advance the understanding of cellular microenvironments. The nanosensor could be utilized for a variety of applications in biomedical research in which the extracellular pH level may be of critical importance, such as cancer research, studies on metabolic disorders, and for diagnostic and treatment applications.

### Supplementary Information

Supplementary Information.

## Data Availability

Data underlying the results presented in this paper can be found in the Supplementary Information or obtained from the corresponding author upon reasonable request.
