# Supplementary material for: A targeted fluorescent nanosensor for ratiometric pH sensing at the cell surface
Source: Sci Rep. 2024 May 29;14:12302. doi: 10.1038/s41598-024-62976-2 (PMC11137054; doi:10.1038/s41598-024-62976-2)
Supplement: Supplementary file 1 — Supplementary Information. [file 41598_2024_62976_MOESM1_ESM.docx]

Supplementary Information for article:

A targeted fluorescent nanosensor for ratiometric pH sensing at the cell surface

Charlotte Kromer^1,2^*, Aaron Katz^1^, Peter Laux^1^, Andreas Luch^1,2^, Harald R. Tschiche^1^

^1^Product Materials and Nanotechnology, Department Chemical and Product Safety, German Federal Institute for Risk Assessment, Berlin, Germany

^2^Institute of Pharmacy, Freie Universität Berlin, Berlin, Germany

* Correspondence:

Charlotte Kromer

Product Materials and Nanotechnology, Department Chemical and Product Safety, German Federal Institute for Risk Assessment, Max-Dohrn-Str. 8-10, 10589 Berlin, Germany

Charlotte.Kromer@bfr.bund.de

**Table A1** Dye and protein loading of the pH_e_ nanosensor.

|  | Loading per mg PS [nmol] |
| --- | --- |
| NR | 5.90 ± 0.58 |
| FITC | 0.848 ± 0.106 |
| WGA | 0.424 ± 0.053 |


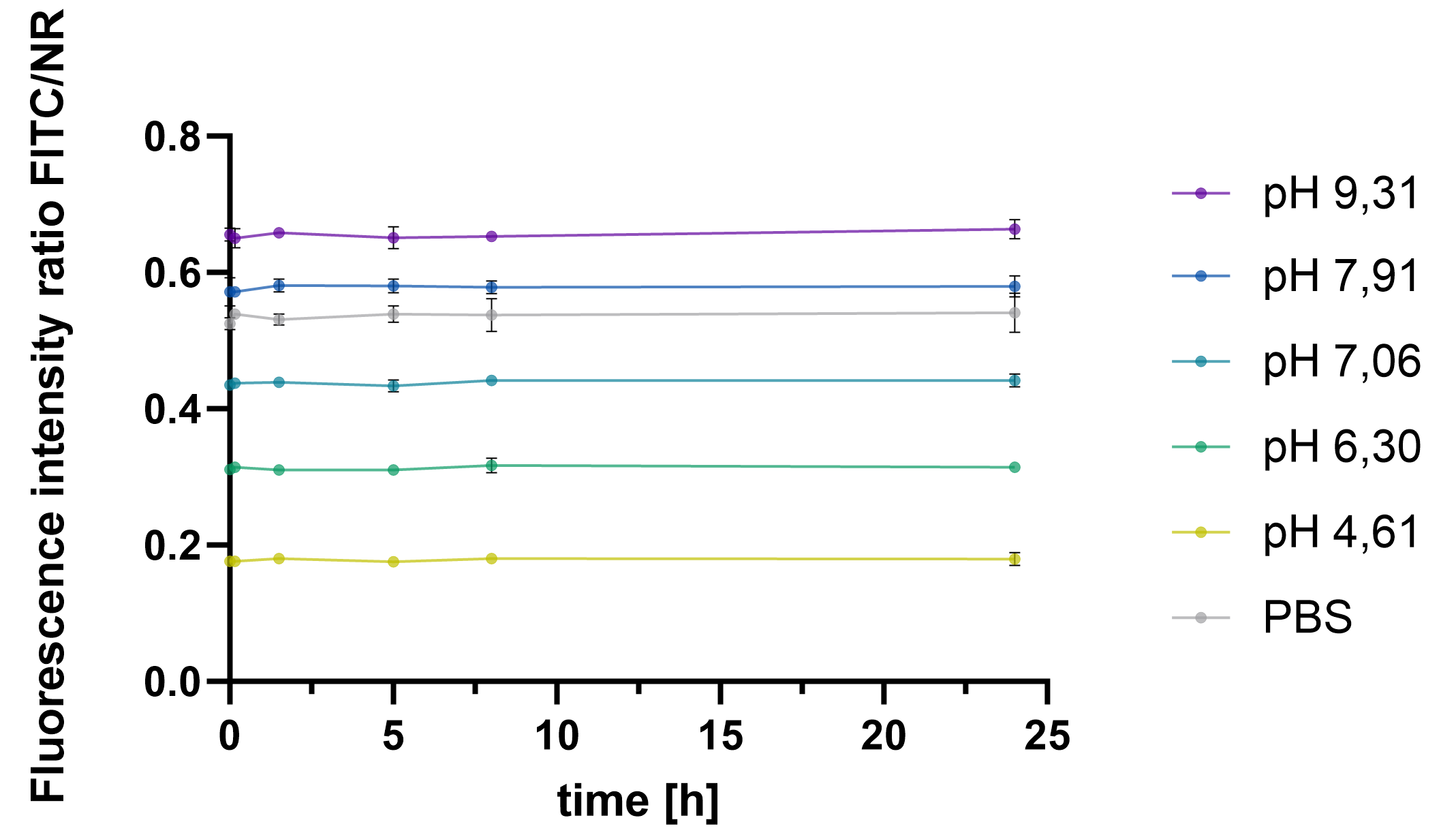


**Figure A1** Evaluation of the long-term stability of the nanosensor at different pH values. BR buffers with 5 different pH values and PBS (pH 7.4) were used. For the data points without error bars, the standard errors were too small to be displayed.


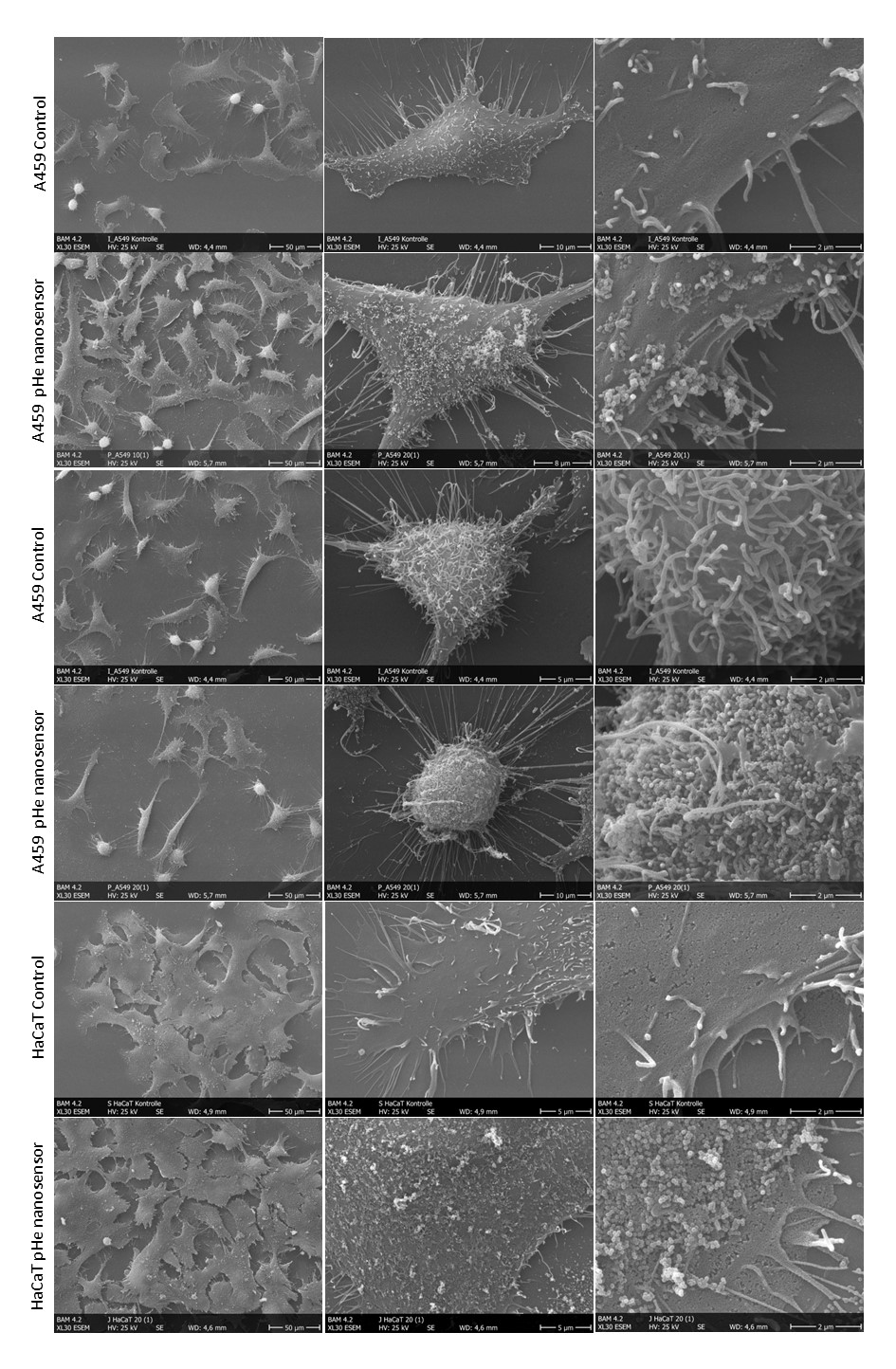


**Figure A2** SEM images of A549 and HaCaT cells at different magnifications. Samples without (control) and with the pH_e_ nanosensor (0.825 mg/ml)


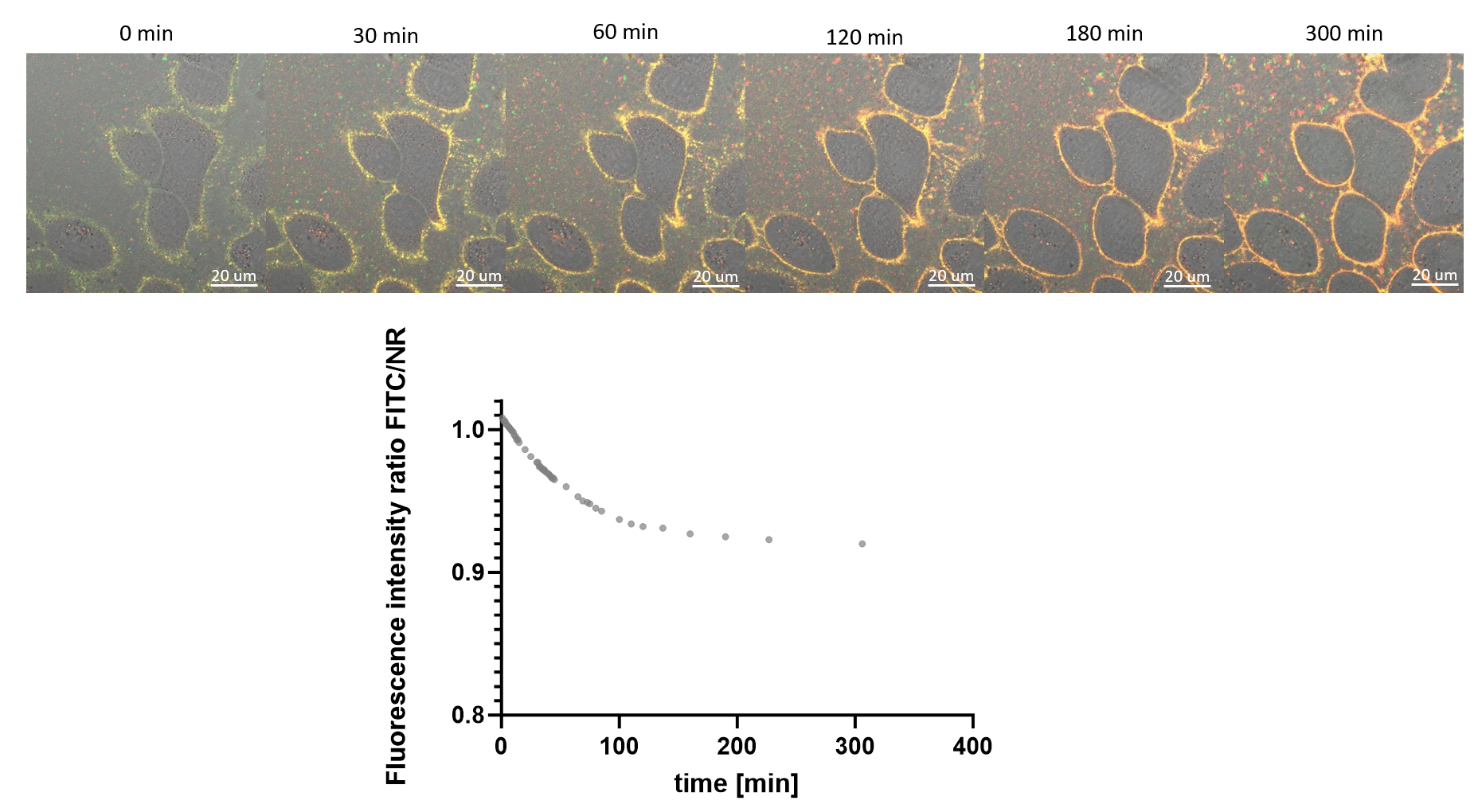


**Figure A3** CLSM images and the derived FI ratio of A549 cells incubated with the nanosensor for 300 min without a washing step.
